# FDG-PET/CT for diagnosis of cyst infection in autosomal dominant polycystic kidney disease

**DOI:** 10.1007/s40336-017-0261-8

**Published:** 2018-02-12

**Authors:** J. P. Pijl, T. C. Kwee, R. H. J. A. Slart, A. W. J. M. Glaudemans

**Affiliations:** 1 Medical Imaging Center, Departments of Nuclear Medicine and Molecular Imaging and Radiology, University of Groningen, University Medical Center Groningen, Groningen, The Netherlands; 2 Department of Biomedical Photonic Imaging, University of Twente, Enschede, The Netherlands

**Keywords:** ADPKD, FDG-PET/CT, Infection

## Abstract

**Purpose:**

Cyst infections are a common complication in autosomal dominant polycystic kidney disease (ADPKD). Diagnosing these infections often remains challenging. Conventional imaging techniques such as ultrasonography, computed tomography (CT), and standard magnetic resonance imaging have several drawbacks and disadvantages. The purpose of this pictorial essay was to illustrate and discuss the potential value of ^18^F-fluoro-2-deoxy-d-glucose positron emission tomography (FDG-PET)/CT in diagnosing cyst infection in ADPKD.

**Methods:**

Exemplary (ADPKD) patients who underwent FDG-PET/CT as part of their routine clinical work-up in our institution are presented to show the potential value and drawbacks of this imaging technique in diagnosing cyst infection. In addition, the current literature and guidelines on this topic were reviewed.

**Results:**

FDG-PET/CT appears to be a sensitive method for the detection of cyst infection, but it is not infallible. Furthermore, FDG uptake in cysts and cyst-like lesions is not specific and clinical and radiological correlations are essential to improve specificity and minimize the risk of falsely discarding other diseases, in particular malignancy.

**Conclusion:**

FDG-PET/CT seems to be a useful imaging modality to diagnose cyst infections in ADPKD. However, its exact diagnostic value has not been established yet due to the lack of a reliable reference standard in previous studies on this topic.

## Introduction

### Autosomal dominant polycystic kidney disease (ADPKD)

ADPKD is the most common hereditary kidney disease that leads to end-stage renal disease [1]. Over the course of years, patients with ADPKD develop multiple cysts in their kidneys. Hepatic cysts are the most common extrarenal manifestation of ADPKD, which can be found in 80% of patients at the age of 30. While ADPKD is often asymptomatic in childhood, progressive expansion of the cysts leads to end-stage renal disease between age 58 and age 80 on average, depending on the causative genetic mutation (PKD1 or PDK2, respectively) [2].

The clinical symptoms of ADPKD are often directly related to the expansion of renal cysts. Patients usually present with complaints of pain, hematuria, but gastrointestinal symptoms such as obstipation and weight loss can also occur [3]. Elevated blood pressure due to increased activity of the renin–angiotensin system is also common [4].

ADPKD is usually diagnosed according to the criteria of Ravine [5], which are based on ultrasonographic imaging and take into account the family history and age of the patient. Patients with a positive family history between the ages of 15 and 29 with more than two renal cysts in total, between 30 and 59 with two cysts in each kidney, or above 60 with more than 4 cysts in each kidney are diagnosed as having ADPKD. Without a positive family history, ADPKD can be diagnosed if more than ten cysts are present in each kidney, regardless of age.

Besides hypertension, end-stage renal disease and pyelonephritis, cyst infection is a common complication of ADPKD [6, 7]. Patients with a cyst infection usually present with symptoms such as flank pain and fever, mostly accompanied by an increased level of C-reactive protein (CRP) and leukocytosis. Because flank pain and fever are also common in cyst hemorrhage, pyelonephritis, and nephrolithiasis, further diagnostic methods are necessary to settle the correct diagnosis [8].

Although cyst infection has a prevalence of up to one in two patients with ADPKD, diagnosis remains challenging [9]. The diagnostic reference is analysis of the cyst fluid, but cyst punctures can cause bleeding, rupture, and contamination of adjacent cysts and are therefore rarely performed [10].

The purpose of this pictorial essay was to illustrate and discuss the potential value of FDG-PET/CT compared to conventional imaging methods (ultrasonography, CT, and MRI) in diagnosing cyst infection in ADPKD.

### Conventional imaging

Ultrasonography is often the initial step in locating the source of lower abdominal pain that is common in patients with cyst infections. It is relatively cheap compared to other cross-sectional imaging methods, does not use any potentially harmful ionizing radiation, and also allows exclusion of hydronephrosis.

However, ultrasonography and other conventional radiologic modalities such as CT and standard magnetic resonance imaging (MRI) are often of limited use in diagnosing infection [11–13]. A diagnosis of infection with these modalities is mostly based on wall thickening and heterogeneous content of the cysts. However, since the presence and expansion of numerous cysts in polycystic disease often severely affects the normal anatomy of the affected organ, wall thickening can reflect residual functional parenchyma, and harmless intracystic cellular debris can also cause a heterogeneous cyst appearance. As a consequence, it is hard to distinguish between infected and non-infected cysts [13].

Besides these limitations, iodinated CT contrast agents and gadolinium-based MRI contrast agents can be contraindicated in patients with impaired renal function (due to ADPKD) because of the potential risks of nephrotoxicity and nephrogenic systemic fibrosis, respectively [13]. Of interest, diffusion-weighted MRI is a relatively new MRI technique that is sensitive to the random (Brownian) motion of water molecules, and can provide a high lesion-to-background contrast. Although it is particularly used in oncology, it may also be used to diagnose (cyst) infections. A recent study showed a sensitivity and specificity around 80% for diagnosing cyst infection with combined MRI findings, which included high intracystic signal intensity on diffusion-weighted imaging MRI and wall thickening. However, only patients with severe cyst infection were included, and the specificity dropped to 66% when patients had organomegaly, which is common in ADPKD [14]. Therefore, there is a need for alternative imaging methods for the evaluation of these patients.

### FDG-PET/CT

^18^F-fluoro-2-deoxy-d-glucose (FDG) positron emission tomography (PET) is considered a potentially useful imaging technique for detecting cyst infection. Activated inflammatory and infectious cells (such as macrophages, lymphocytes and neutrophils) require a high amount of glucose and therefore also accumulate FDG [15]. The FDG accumulation can be visualized by PET with a high contrast-to-background ratio, and the concomitantly acquired low-dose CT can be used to anatomically pinpoint the sites of FDG accumulation and therefore sites of potential infection.

**Fig. 1:**
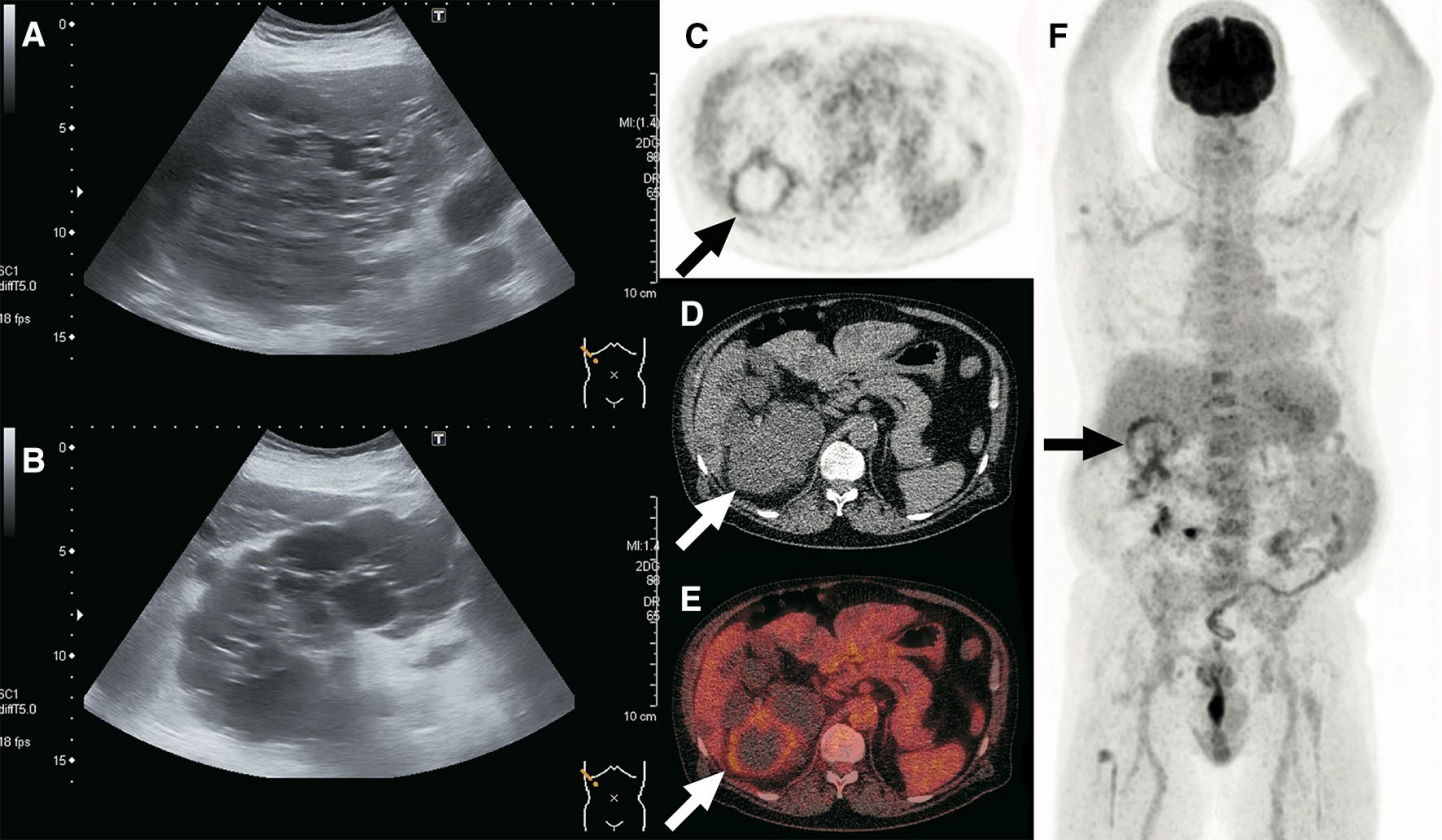
A 61-year-old man with ADPKD and unilateral renal agenesis was admitted to the hospital because of a possible urinary tract infection. He presented with complaints of cold shivers, pollakiuria, stranguria, lower abdominal pain and cloudy urine. Blood tests showed a CRP level of 363 mg/L and a white blood cell count of 6.6 × 10^9^/L, while a urine culture tested positive for *Escherichia coli*. The patient’s clinical condition improved under ciprofloxacin treatment, but his CRP level remained high 5 days later at 154 mg/L. Because of the possibility of pyelonephritis or cyst infection, ultrasound was ordered. Ultrasonographic images of the liver (**a**) and kidney (**b**) showed multiple cysts, but no hydronephrosis and no potential site of infection. Subsequently, FDG-PET/CT was performed. Axial FDG-PET (**c**), low-dose CT (**d**), fused FDG-PET/CT (**e**), and coronal maximum intensity projection FDG-PET (**f**) showed pathologic FDG uptake of the wall of a cyst in the right kidney (arrows), consistent with cyst infection. No pathologic FDG-avid foci were detected elsewhere. The patient was discharged 1 day after the FDG-PET/CT scan in good clinical condition, where he continued his course of ciprofloxacin for 10 more days

**Fig. 2:**
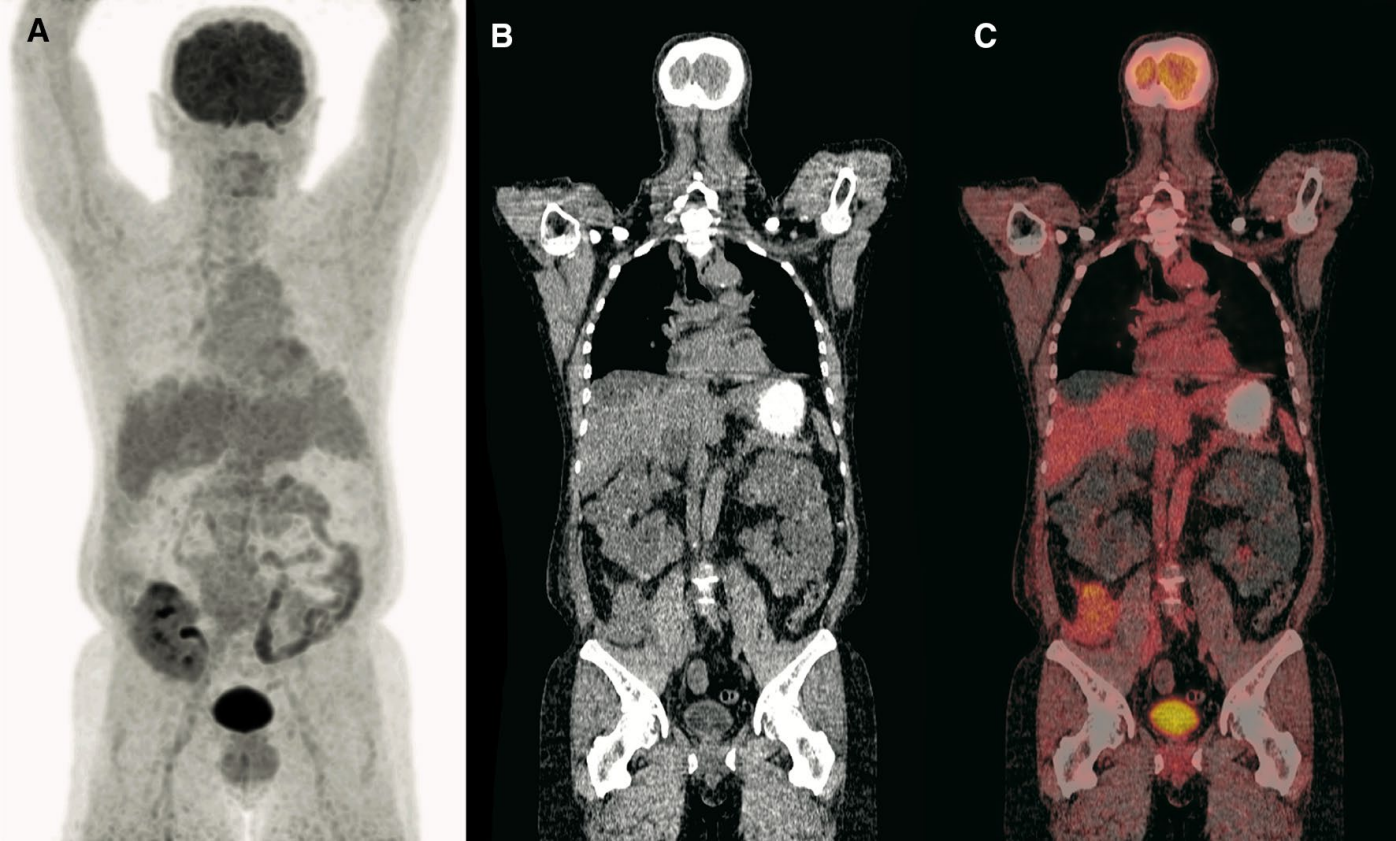
A 53-year-old man with ADPKD presented at the emergency department because of abdominal pain, fever and a possible urinary tract infection. He had received a kidney transplant 1 month before because of end-stage renal disease. Blood tests showed a CRP level of 19 mg/L and a white blood cell count of 8.6 × 10^9^/L, while a urine culture revealed Gram-negative rods. The patient was admitted, and an FDG-PET/CT scan was ordered to assess the native kidneys for infection. Coronal maximum intensity projection FDG-PET (**a**), low-dose CT (**b**), and fused FDG-PET/CT (**c**) did not show any signs of (cyst) infection in the native kidneys, and no pathologic FDG-avid foci elsewhere. FDG-PET/CT findings provided confidence to the clinicians to attribute the clinical and laboratory findings to a simple cystitis. The patient was sent home in good clinical condition with a course of nitrofurantoin for 3 days

**Fig. 3:**
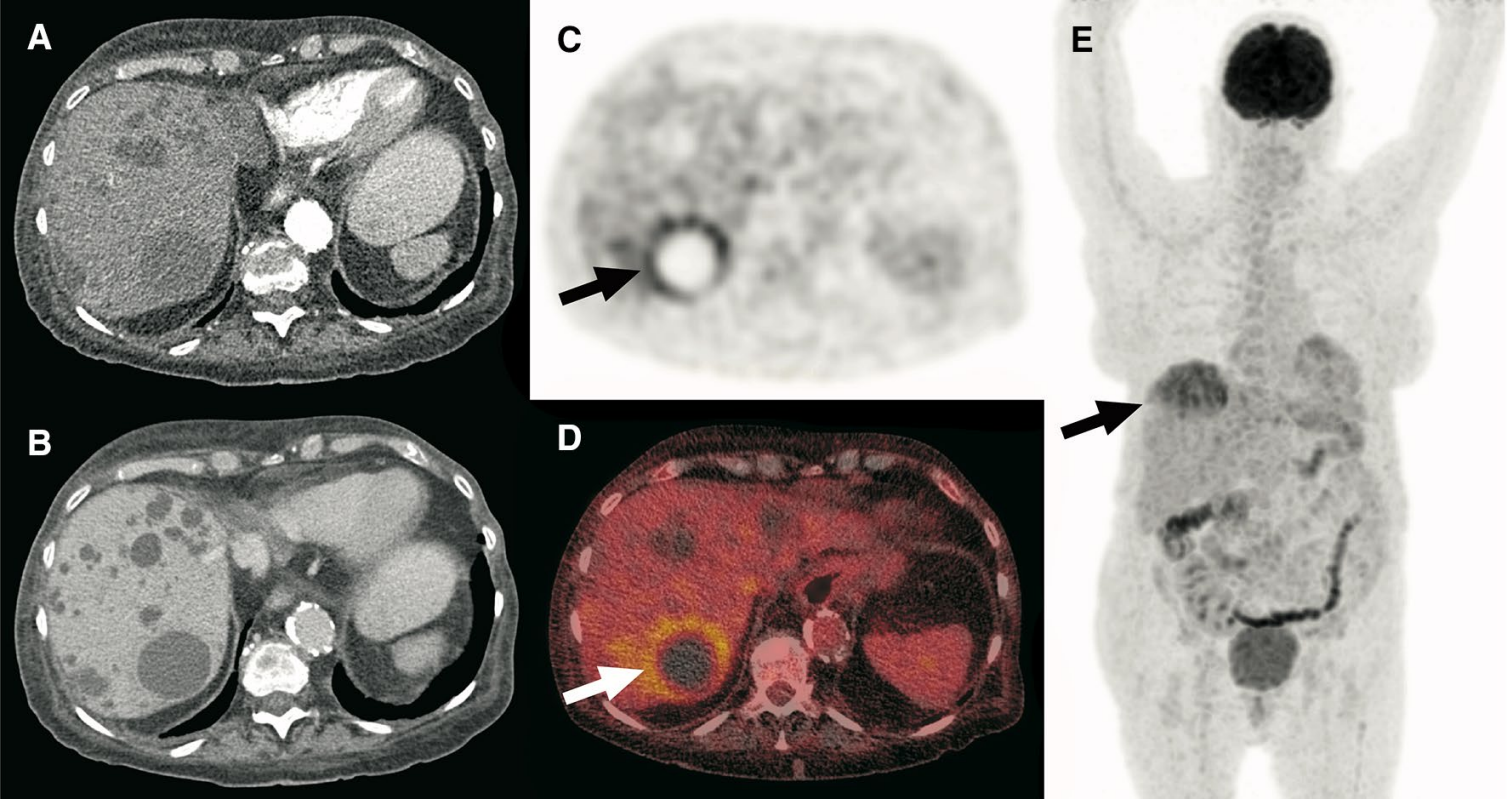
A 68-year-old woman with ADPKD presented with a feeling of general malaise that had been increasing over the course of 2 weeks. Nocturnal sweats were also present in the last week. There was no fever or abdominal pain. Blood tests showed a CRP level of 242 mg/L and a white blood cell count of 15.8 × 10^9^/L. Blood cultures were positive for *Escherichia coli* and urine cultures were positive for *Klebsiella pneumoniae*. The patient was admitted with a differential diagnosis of urosepsis or an infection of her abdominal aortic endograft. Despite intravenous treatment with cefuroxime for 4 days, infectious parameters remained high (CRP level of 228 mg/L). To find the source of infection, a contrast enhanced CT scan of the abdomen was ordered. Axial arterial phase (**a**) and portal-venous phase (**b**) full-dose CT scans showed multiple liver cysts that were otherwise unremarkable. Because CT failed to identify any site of infection, an FDG-PET/CT scan was ordered. Axial FDG-PET (**c**) fused FDG-PET/CT (**d**), and coronal maximum intensity projection FDG-PET (**e**) showed pathologic FDG uptake of the wall of a cyst in segment seven of the liver (arrows), in keeping with cyst infection. No pathologic FDG-avid foci were detected elsewhere. The antibiotic regimen was changed to ceftriaxone for better cyst penetration. CRP levels decreased to 49 mg/L in 4 days. The patient was sent home in good condition with a subsequent course of ciprofloxacin

**Fig. 4:**
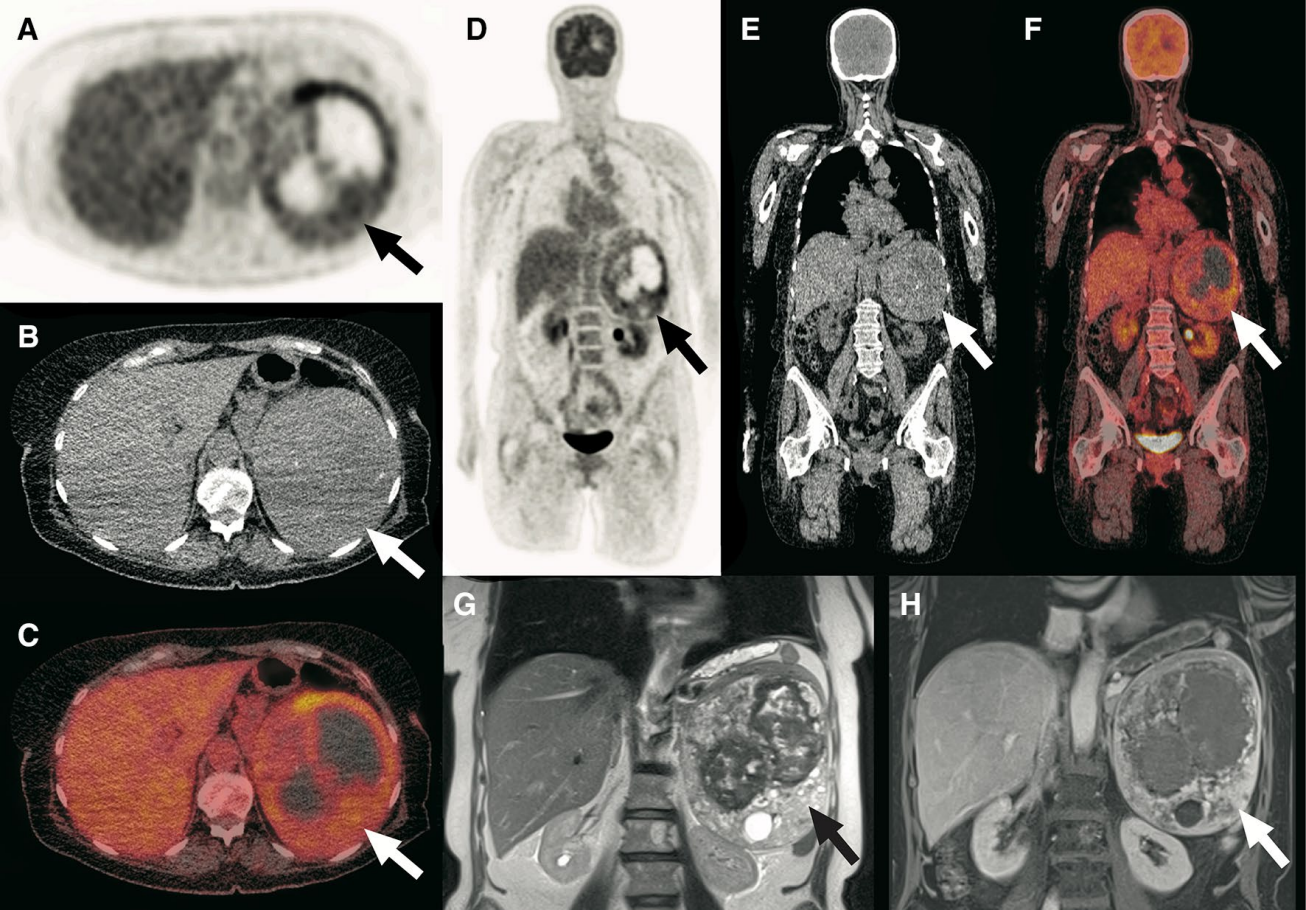
A 71-year-old woman presented to the rheumatology and immunology department because of fatigue, general malaise and involuntary weight loss of 9 kg in 5 months. She did not have fever or abdominal pain. Blood tests showed a CRP level of 256 mg/L and a white blood cell count of 11.0 × 10^9^/L. An FDG-PET/CT scan was ordered to assess the possibility of vasculitis, infection, or malignancy. Axial FDG-PET (**a**), low-dose CT (**b**), and fused FDG-PET/CT (**c**) showed an abnormal spleen (arrows) that was markedly enlarged with inhomogeneous areas of pathologic FDG uptake and central photopenic areas that could not be clearly assessed on the low-dose CT (**b**). Coronal FDG-PET (**d**), low-dose CT (**e**), and fused FDG-PET/CT (**f**) demonstrated similar findings (arrows). No pathologic FDG-avid foci were detected elsewhere. Based on the laboratory and FDG-PET/CT findings, splenic cyst infection was strongly considered, although other conditions (in particular malignancy) could not be excluded. MRI was ordered for further assessment of the spleen. Coronal T2-weighted (**g**) and gadolinium-enhanced fat-suppressed T1-weighted (**h**) images showed a markedly enlarged and inhomogeneous spleen (arrows) with cyst-like changes, and centrally both T2 hypointense and hyperintense non-enhancing components. The differential diagnosis based on the MRI examination included hemangioma with hemorrhage, infected splenic cyst with abscess formation, and angiosarcoma. Splenectomy was performed 1 day later, which demonstrated angiosarcoma with central hemorrhagic areas

**Fig. 5:**
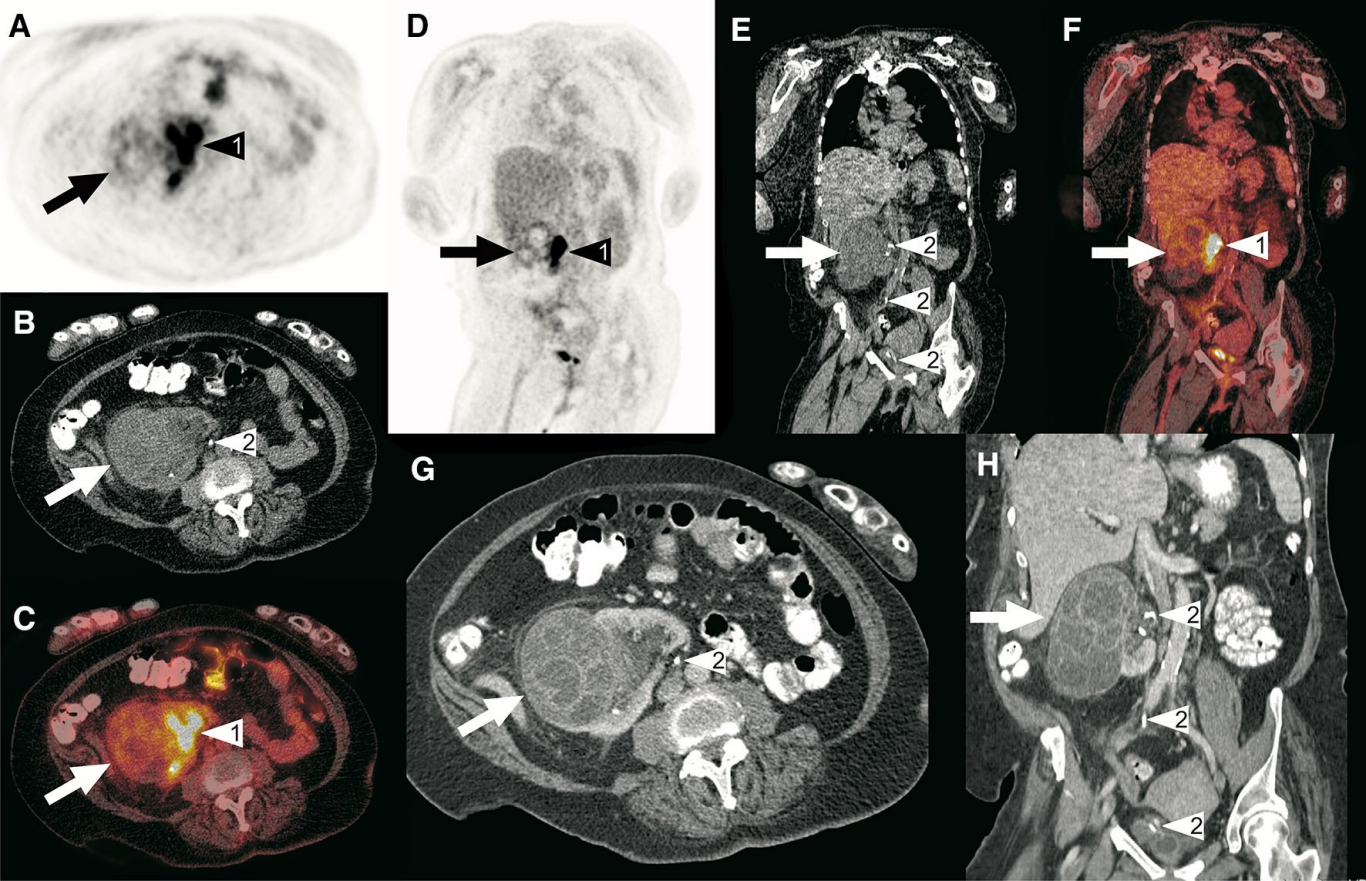
A 62-year-old woman was admitted to the department of internal medicine because of abdominal pain and fever. A couple of weeks before, she was diagnosed with a large obstructing right ureteral stone, for which a double-J catheter was placed. Blood and urine samples were taken, which both tested positive for *Proteus mirabilis*. The CRP level was 65 mg/L and the white blood cell count was 12.4 × 10^9^/L. The working diagnosis was urosepsis and the patient was started on ciprofloxacin and amoxicillin/clavulanic acid, which was later switched to meropenem. Despite antibiotic treatment, high infectious parameters (CRP level of 101 mg/L and white blood cell count of 13.8 × 10^9^/L) and fever remained. To assess for any underlying cause of infection, an FDG-PET/CT scan was ordered. Axial and coronal FDG-PET (**a**, **d**), low-dose CT (**b**, **e**) and fused FDG-PET/CT (**c**, **f**) show an enlarged contour of the right kidney that appeared cystic, but with only slightly increased FDG uptake (arrows). Because of the slightly increased FDG uptake, infection was deemed unlikely. No pathologic FDG-avid foci were detected elsewhere. Axial (**g**) and **h** portal-venous phase full-dose CT scans showed a multi-cystic lesion in the cortex of the right kidney (arrows), with a differential diagnosis that included cyst infection and cystic neoplasm. Also note normal pyelocaliceal FDG accumulation in the right kidney (arrowheads no. 1 in **a**, **c**, **d**, and **f**) and the double-J catheter (arrowheads no. 2 in **b**, **e**, **g**, and **h**). Because the patient had already been treated with high-dose intravenous antibiotics for a long time without clinical improvement, a nephrectomy was performed, which demonstrated a necrotizing and suppurative infection of multiple cysts, without any signs of malignancy. The patient was discharged from the hospital in good clinical condition shortly afterwards

Various recent studies have investigated the diagnostic value of FDG-PET/CT in diagnosing cyst infections. For example, Sallée et al. [16] described 8 patients in whom a cyst infection was diagnosed based on FDG-PET/CT. Five patients had a definite cyst infection based on cyst aspiration, and three patients a probable cyst infection based on the presence of all of the following five factors: fever, abdominal pain, increased CRP, the absence of cyst bleeding and the absence of any other possible cause of fever. In all 8 patients, FDG-PET/CT results were positive for infection (Figs. 1, 2, 3, 4 and 5).

Bobot et al. [10] described 32 cases of clinically suspected cyst infections in 24 patients where FDG-PET/CT achieved a sensitivity of 77% and a specificity of 100% for diagnosing cyst infection. Three FDG-PET/CT positive cases were regarded as definitive cyst infection based on cyst aspiration, the other 11 FDG-PET/CT positive cases were considered as ‘likely cyst infections’ based on the five criteria of Sallée et al. [16]. Long treatment with antibiotics before the FDG-PET/CT scan was suggested as the most likely explanation for false-negative cases.

Balbo et al. [17] described 32 cases of clinically suspected cyst infections in 27 patients where FDG-PET/CT was reported to have a sensitivity of 95% and a specificity of 100%. Among these, there were 24 episodes of cyst infection in 18 patients. Six cases were definite cyst infections based on cyst aspiration, the other 18 cases were ‘probably cyst infections’, also based on the criteria of Sallée et al. [16].

Finally, we recently analyzed 37 cases in 32 patients where FDG-PET/CT achieved a sensitivity of 86% and a specificity of 81%, with 18 true-positive and 11 true-negative cases (data not published yet). All cases were assessed based on the criteria of Sallée et al. [16], because no cyst punctures were performed.

## Discussion

Cyst infections are often a diagnostic challenge, particularly in case of polycystic disease such as ADPKD. We reviewed the current evidence and provided exemplary cases to illustrate the potential benefits and pitfalls of FDG-PET/CT in diagnosing cyst infection.

In cases 1 and 3, the correct diagnosis of a cyst infection was based on a positive FDG-PET/CT scan, and in case 2 a suspected cyst infection was ruled out. In cases 1 and 3, the patients clinically improved on antibiotics able to penetrate cyst walls, and in case 2, the patient improved on a course of nitrofurantoin for cystitis. All patients were discharged from hospital in good clinical condition.

Case 4 illustrated that FDG-PET/CT can also result in a false positive diagnosis of a cyst infection in more complicated cysts. Increased FDG uptake in complicated cyst-like structures is not specific for infection and can also reflect malignancy [18]. This was also shown in case 5, where a complicated cyst (Bosniak category 3–4) did not show markedly increased FDG uptake, although pathologic testing later confirmed the presence of multiple infected cysts.

In the current guidelines for use of FDG-PET/CT in inflammation and infection, several diseases are marked as ‘major indication’ based on sufficient evidence in literature of FDG-PET/CT’s high sensitivity and specificity in these diseases [19]. These include, for example, sarcoidosis, spinal infection, and vasculitides. Other useful applications that are mentioned, but are not a major indication yet due to insufficient evidence, include evaluation of suspected infection of intravascular devices and potentially infected hepatic and renal cysts in polycystic diseases.

These guidelines, however, were established in 2013 based on 7 studies reporting in total only 34 scans in 28 patients [20–26]. Since then, various larger studies were published, including in total more than a hundred cases and describing in all cases a high sensitivity and specificity for FDG-PET/CT in diagnosing cyst infection ([10, 17], own study (not published yet)). Therefore, it seems appropriate to reassess the guidelines and to state whether a suspected cyst infection in polycystic diseases should be considered a major indication for performing an FDG-PET/CT scan.

However, the main limitation of these recent studies [10, 16, 17] was the lack of a gold diagnostic standard to validate the results of the FDG-PET/CT scan. As mentioned previously, cyst puncture is currently the only true gold diagnostic standard, but it may lead to serious complications and is therefore rarely performed. The majority of the cases that were considered as true positives were diagnosed based on the five clinical criteria from Sallée et al. [16]. Therefore, patients who missed one or more of the clinical criteria from Sallée et al. [16] but did show signs of cyst infection on the FDG-PET/CT scan were considered as false positives. Patients who met all five criteria but did not show signs of infection on the FDG-PET/CT scan, were considered as false negatives. The possibility that at least some patients were suffering from a cyst infection without showing all clinical criteria from Sallée et al. seems plausible [16].

In conclusion, FDG-PET/CT is a potential imaging modality to diagnose cyst infections in ADPKD. However, due to the lack of a solid reference standard, most studies in this field relied on a combination of clinical factors to diagnose cyst infection, which is which is a far from optimal method to validate a new diagnostic tool to validate a new diagnostic tool.
